# HIV-exposed children account for more than half of 24-month mortality in Botswana

**DOI:** 10.1186/s12887-016-0635-5

**Published:** 2016-07-21

**Authors:** Rebecca Zash, Sajini Souda, Jean Leidner, Heather Ribaudo, Kelebogile Binda, Sikhulile Moyo, Kathleen M. Powis, Chipo Petlo, Mompati Mmalane, Joe Makhema, Max Essex, Shahin Lockman, Roger Shapiro

**Affiliations:** Beth Israel Deaconess Medical Center, 110 Francis Street, Suite GB, Boston, MA 02215 USA; Botswana Harvard AIDS Institute Parternship, Private Bag BO320, Gaborone, Botswana; Harvard T.H. Chan School of Public Health, 651 Huntington Ave, Boston, MA 02115 USA; University of Botswana, Faculty of Health Sciences, Gaborone, Botswana; Goodtables Data Consulting, Oklahoma, USA; Massachusetts General Hospital, 55 Fruit St, Boston, MA 02114 USA; Ministry of Health, Gaborone, Botswana; Harvard Medical School, Boston, MA USA; Brigham and Women’s Hospital, Infectious Diseases Division, 75 Francis Street, Boston, MA 02115 USA

**Keywords:** HIV-exposed uninfected, Child mortality, Breastfeeding, HIV-infected children, PMTCT, Sub-Saharan Africa

## Abstract

**Background:**

The contribution of HIV-exposure to childhood mortality in a setting with widespread antiretroviral treatment (ART) availability has not been determined.

**Methods:**

From January 2012 to March 2013, mothers were enrolled within 48 h of delivery at 5 government postpartum wards in Botswana. Participants were followed by phone 1–3 monthly for 24 months. Risk factors for 24-month survival were assessed by Cox proportional hazards modeling.

**Results:**

Three thousand mothers (1499 HIV-infected) and their 3033 children (1515 HIV-exposed) were enrolled. During pregnancy 58 % received three-drug ART, 23 % received zidovudine alone, 11 % received no antiretrovirals (8 % unknown); 2.1 % of children were HIV-infected by 24 months. Vital status at 24 months was known for 3018 (99.5 %) children; 106 (3.5 %) died including 12 (38 %) HIV-infected, 70 (4.7 %) HIV-exposed uninfected, and 24 (1.6 %) HIV-unexposed. Risk factors for mortality were child HIV-infection (aHR 22.6, 95 % CI 10.7, 47.5 %), child HIV-exposure (aHR 2.7, 95 % CI 1.7, 4.5) and maternal death (aHR 8.9, 95 % CI 2.1, 37.1). Replacement feeding predicted mortality when modeled separately from HIV-exposure (aHR 2.3, 95 % CI 1.5, 3.6), but colinearity with HIV-exposure status precluded investigation of its independent effect. Applied at the population level (26 % maternal HIV prevalence), an estimated 52 % of child mortality occurs among HIV-exposed or HIV-infected children.

**Conclusions:**

In a programmatic setting with high maternal HIV prevalence and widespread maternal and child ART availability, HIV-exposed and HIV-infected children still account for most deaths at 24 months. Lack of breastfeeding was a likely contributor to excess mortality among HIV-exposed children.

## Background

The HIV pandemic has profoundly decreased child survival in sub-Saharan Africa [1–4]. Improvements in strategies to prevent mother-to-child HIV infection (PMTCT) partially reversed this trend, but a shift to formula feeding as part of PMTCT efforts may have eroded mortality gains related to pediatric HIV reduction [5, 6]. Additionally, HIV exposed but uninfected (HEU) children have a known increased risk of death compared with HIV-unexposed (HU) children [7–13], whether formula fed or breastfed [14–19].

Botswana, an African country where under-five mortality almost doubled from 1990 to 2000 due to HIV [4], began to provide free formula to all HIV-exposed infants from birth in 1999 and started the rollout of antiretroviral therapy (ART) for HIV-infected adults and children in 2001. Beginning in 2012, all pregnant women had access to 3-drug ART for PMTCT. These PMTCT and ART program changes have occurred without documentation of accurate child mortality rates and the relative contributions of HIV-infected and HEU children to overall 24-month child mortality remain unknown.

Current methods to estimate childhood mortality typically underestimate deaths in areas with high HIV prevalence [20], and clinical trials may similarly underestimate the true population rate because of increased care and resources within a trial. At the request of the Botswana Ministry of Health and with the support of the United States Centers for Disease Control and Prevention (CDC), we performed a prospective observational study to obtain accurate child mortality rates, accounting for HIV status and infant feeding practices, to better understand overall risk and identify modifiable risk factors for child mortality in Botswana. Our study followed a cohort of mothers and infants for 24 months post-partum using telephone follow up in order to have minimal impact on clinical outcomes.

## Methods

An equal number of HIV-infected and HIV-uninfected mothers and their infants were enrolled at five geographically diverse public hospital maternity wards throughout Botswana: Deborah Retief Memorial Hospital (DRM) in Mochudi, Nyangabwe Referral Hospital (NH) in Francistown, Letsholathebe Memorial Hospital (LMH) in Maun, Bamalete Lutheran Hospital (BLH) in Ramotswa and Seventh Day Adventist Hospital (SDA) in Kanye. These hospitals were chosen to maximize geographic diversity but maintain adequate number of deliveries to reach recruitment goals and include one tertiary referral center (NRH), one district hospital, and three regional hospitals. In Botswana, approximately 95 % of women delivery at maternity wards [21].

We systematically approached all HIV-infected mothers and the same number of HIV-uninfected mothers on the post-partum ward for enrollment in our study at each site during working hours (M-F 7:30-4:30). Mothers were excluded if they lived >100 km away, were unwilling to be contacted by telephone, or could not provide a contact number (mothers without their own phones were encouraged to provide a number for a relative, friend, neighbor or workplace).

We conducted follow up visits via telephone at one and three months, then every three months until 24 months post-partum. Maternal demographics, medical and obstetric history and HIV testing and treatment history were collected at baseline. During follow up we assessed vital status of both mother and child, information about child feeding method and vaccinations, child and maternal hospitalizations, food security, medications, time child and mother were apart and HIV testing. Participants who could not be contacted by phone were visited at home (if they had provided permission). Mothers were followed until death, completion of study or death of their infant. Infants were followed until death or completion of the study, regardless of maternal survival.

Study participants received routine care through Botswana government providers, and HIV-infected mothers and children were eligible to receive ART and PMTCT according to Botswana national HIV guidelines (free for Botswana citizens). During our study guidelines changed: from February- May 2012, pregnant HIV-infected women with CD4 ≤ 350 cells/mm^3^ or WHO clinical stage three or four could start combination ART (primarily ZDV/lamivudine[3TC]/Nevirapine[NVP]) to be continued life-long and pregnant women with CD4 counts >350 cells/mm^3^ were eligible to start ZDV alone during pregnancy. From June 2012, all pregnant women were eligible to start ART with Atripla (tenofovir/emtricitabine/efavirenz) at government HIV clinics. Guidelines recommended mothers with CD4 ≤ 350 cells/mm^3^ continue ART life-long [22].

HIV-exposed infants were tested for HIV in the government system at six weeks by PCR and 18 months by ELISA. As part of the study, dried blood spots (DBS) were collected among all HIV-exposed infants at enrollment and selectively tested by PCR at the end of the study if no other HIV testing was available or if HIV infection was reported. HIV-infected children were eligible to receive ART at the time of diagnosis which generally consisted of lopinavir/ritonavir/ZDV/3TC.

### Feeding

In Botswana, HIV-uninfected mothers are counseled to breastfeed exclusively. HIV-infected mothers are counseled to exclusively formula feed and receive free formula, though HIV treatment guidelines were updated in June 2012 to allow mothers receiving ART to choose to breastfeed. At each follow up encounter mothers were asked about infant feeding practices (breastfeeding, formula feeding, supplementary water and/or solid foods). For analysis we categorized feeding as ever vs. never breastfed. Among infants surviving at least 28 days, we also compared breastfeeding for at least one month vs. all others (breastfeeding less than one month or never breastfeeding).

### Child and maternal outcomes

Children were considered HIV-infected by any positive HIV PCR test or a positive HIV ELISA after 17 months. Exclusively formula fed children were considered HIV-uninfected if they had a negative HIV-test after four weeks, while breastfed children required a negative HIV test at least four weeks after weaning. In the primary analysis, HIV-exposed children with unknown infection status were considered HIV-uninfected. Since early mortality may have precluded determination of HIV-serostatus, additional sensitivity analyses were performed that 1) assumed HIV-unknown children who died were HIV-infected and 2) assumed HIV-unknown children who were “high risk” were HIV-infected. “High-risk” was defined as children whose mothers had < four weeks of ARVs (including no ARVs) in pregnancy.

Child deaths, maternal deaths, and the date of death were reported by the primary caregiver reached at follow-up. Verbal autopsy was performed for children who died using structured interviews. The cause of death was categorized by consensus of physician members of the study team (blinded to all information except verbal autopsy).

Projections of 24-month child mortality in Botswana were calculated by applying the cumulative mortality rate for HIV-infected, HEU and HU infants found in this study to hypothetical populations of children made by varying maternal HIV prevalence from 5 to 30 % and MTCT rates from 0 to 20 %.

### Statistical analysis

Cumulative child mortality was calculated from participants with complete follow up and mortality rate is presented as deaths per 1000child- years. Risk factors for mortality were assessed using Cox proportional hazards modeling for all children and separately for HEUs. Time-varying risk factors were feeding (BF vs. no BF), receipt of medications, separation from caregiver, introduction of solid food, food insecurity, and maternal vital status. Multivariate models included non-collinear covariates with a *p*-value <0.20. Maternal age and CD4 count (in HEU model) were also included due to previously established association with child mortality [12, 23]. Infants found to be HIV-infected at their six-week PCR were assumed infected *in utero* or peri-partum and therefore considered HIV-infected for the entire study. However, because timing of seroconversion was uncertain we also separately modeled early mortality (<43 days) by HIV-exposure status alone and late mortality (≥43 days) by both HIV-exposure and HIV-infection status. All tests were two tailed and *p*-values of <0.05 were considered statistically significant. Statistical analyses were performed with SAS, version 9.4 (SAS Institute, Cary, North Carolina).

## Results

Between January 2012 and March 2013 we enrolled 3000 mothers (1499 HIV-infected and 1501 HIV-uninfected) and their 3033 infants (1515 HIV-exposed and 1518 HIV-unexposed). This represented 21 % of all deliveries, and 37 % of all HIV-infected deliveries at participating sites. Enrollment corresponded to size of the delivery ward, with 46 % of participants enrolled in Francistown, 28 % in Maun, 11 % in Kanye, 9 % in Mochudi, and 6 % in Ramotswa. HIV-infected mothers tended to be older, multi-gravid and have lower educational attainment than HIV-uninfected mothers (all *p* < 0.001) (Table 1). HIV-infected mothers were also less likely to have an indoor toilet and water piped directly into the house (*p* < 0.001). Infants born to HIV-infected women were more likely to be born preterm (<37 weeks gestational age) and low birthweight (<2500 g) (Table 1). All infants were discharged alive from the hospital.

**Table 1:** Maternal and infant baseline characteristics

Maternal characteristics	HIV-infected (*N* = 1499)	HIV-uninfected (*N* = 1501)
Age (median)	29	24
Primigravid	244 (16 %)	675 (45 %)
Gravidity ≥4	545 (36 %)	199 (13 %)
Married	122 (8 %)	145 (10 %)
Education		
Primary/None	197 (13 %)	77 (5 %)
Secondary	1176 (78 %)	1127 (75 %)
Tertiary	126 (8 %)	297 (20 %)
Drinking water source		
Communal standpipe	301 (20 %)	170 (11 %)
Tap in yard	977 (65 %)	1007 (67 %)
Piped directly into the home	209 (14 %)	317 (21 %)
Other/Unknown	12 (1 %)	7 (0.5 %)
Toilet facilities		
No latrine facilities	107 (7 %)	60 (4 %)
Shared latrine with other compounds	69 (5 %)	53 (4 %)
Private latrine for house/compound	1028 (69 %)	946 (63 %)
Indoor Toilet	289 (19 %)	434 (29 %)
Other/Unknown	6 (0.4 %)	8 (1 %)
Electricity in the home	799 (53 %)	987 (66 %)
VDRL positive during pregnancy	32 (2 %)	25 (2 %)
1st Hemoglobin during pregnancy (median)	10.8 g/dl	11.3 g/dl
Infant characteristics	HIV-exposed (*N* = 1515)	HIV-unexposed (*N* = 1518)
Gender		
Male	747 (49 %)	775 (51 %)
Female	768 (51 %)	743 (49 %)
Delivery method		
Vaginal	1334 (88 %)	1387 (91 %)
Elective Cesarean section	21 (1 %)	16 (1 %)
Emergency Cesarean section	160 (11 %)	115 (8 %)
Gestational age at Delivery		
Very preterm (≤32 week)	44 (3 %)	12 (1 %)
Preterm (33–37 weeks)	233 (15 %)	180 (12 %)
Term (≥37 weeks)	1228 (81 %)	1316 (87 %)
Unknown	10 (1 %)	10 (1 %)
Birthweight		
Very low birthweight (≤1500 g)	2 (0.1 %)	2 (0.1 %)
Low birthweight (1500–2500 g)	252 (17 %)	111 (7 %)
Normal birthweight (>2500 g)	1261 (83 %)	1405 (93 %)
Required NNU	50 (3 %)	33 (2 %)

The vast majority of HIV-unexposed infants were breastfed (*N* = 1513, 99.7 %), with 99 % (*N* = 1505) having breastfed for at least one month and 75 % (*N* = 1130) for more than six months. In contrast, only 16 % (*N* = 249) of HIV-exposed infants were ever breastfed;13 % (*N* = 195) for at least one month and 1.4 % (*N* = 21) for more than six months. Less than one-third of HIV-infected infants (*N* = 9, 28 %) were ever breastfed. The timing of the introduction of solid food was similar between HIV-exposed and HIV-unexposed infants; solid foods were reported in 74 % of HU infants and 71 % of HIV-exposed infants by six months of age.

### Infant HIV infection

Mother to child transmission of HIV at 24 months was 2.1 % and did not differ by site. One hundred twenty children (8 %) had unknown HIV-status at 24-months; 44 (2.9 %) died before receiving an HIV test, 42 (2.8 %) exclusively formula-fed children were never tested at or after one month of age and 34 (2.2 %) children were never tested after cessation of breastfeeding.

### Hospitalizations

Overall, 414 (14 %) children were hospitalized at least once during follow-up, including 170 (11 %) HU, 230 (16 %) HEU, and 14 (44 %) HIV-infected children (*p* < 0.001). HEU children were 1.6 times more likely than HU children to spend >3 weeks in the hospital (RR 1.6, 95 % CI 0.8,3.4), though this result was not statistically significant. Of the 14 HIV-infected children who were hospitalized at least once during follow-up, 75 % were in hospital for one to three weeks.

### 24-month child mortality

We obtained vital status on 3018 (99.5 %) children at 24 months. Nine children withdrew from the study and six were lost to follow up. Overall, 106 children died, yielding a cumulative mortality of 3.5 % (18 deaths per 1000 child-years). The cumulative probability of death was 38 % (*N* = 12) among HIV-infected children (300 deaths per 1000 child-years), 4.7 % (*N* = 70) among HEUs (24 deaths per 1000 child-years), and 1.6 % (*N* = 24) among HUs (8 deaths per 1000 child-years). There was no significant difference in mortality by site. Almost one-third (30 %) of deaths occurred in the first 42 days of life, including 28 (34 %) among HIV-exposed infants (regardless of infection) and four (17 %) among HIV-unexposed infants.

Cause of death is shown in Table 2 and did not differ significantly by HIV-exposure status (*p* = 0.25). In the 4 weeks prior to death, 90 (85 %) children were seen by a healthcare provider, including 82 (77 %) who sought care from a clinic or hospital, 3 (3 %) who were evaluated by a traditional healer and 8 (8 %) seen by both. Of the 14 children with no contact with the health care system prior to death, four (29 %) died from accident/trauma.

**Table 2:** Cause of death and season of death by child HIV status

	Total deaths (*N* = 106)	HIV-infected deaths (*N* = 12)	HIV-exposed uninfected deaths (*N* = 70)	HIV-unexposed deaths (*N* = 24)
Cause of Death (N, % of total deaths)				
Diarrheal Illness	46 (43 %)	6 (50 %)	32 (46 %)	8 (33 %)
Pneumonia	14 (13 %)	1 (8 %)	9 (13 %)	4 (17 %)
Respiratory Illness	17 (16 %)	3 (25 %)	10 (14 %)	4 (17 %)
Other Infection	8 (8 %)	1 (8 %)	6 (9 %)	1 (4 %)
Trauma/Accident	5 (5 %)	0	2 (3 %)	3 (13 %)
Other (non-infectious)	5 (5 %)	0	3 (4 %)	2 (8 %)
Unable to classify	11 (10 %)	1 (8 %)	8 (11 %)	2 (8 %)
Death by season of Birth (N, % of total births who died)^a^				
Jan-March (Rain, hot) (*N* = 995 total births)	50 (5 %)	6 (46 %)	31 (6 %)	13 (3 %)
Apr-June (Dry, temperate) (*N* = 1009 total births)	34 (3 %)	3 (30 %)	24 (5 %)	7 (1 %)
July–Sept (Dry, cool) (*N* = 631 total births)	11 (2 %)	3 (38 %)	6 (2 %)	2 (1 %)
Oct–Dec (Variable rain, hot) (*N* = 415 total births)	11 (3 %)	0 (0 %)	9 (5 %)	2 (1 %)

^a^The % of deaths by season of birth was calculated as the number of deaths/total number of births in each time period. Among HIV-infected children there were 13 births in Jan–Mar, 10 in April–June, 8 in July–Sept and 1 in Oct–Dec. Among HIV-exposed uninfected children there were 505 births in Jan–Mar, 498 in April–June, 287 in July–Sept and 193 in Oct–Dec. Among HIV-unexposed children there were 477 births in Jan–Mar, 501 in April–June, 319 in July–Sept and 221 in Oct–Dec

**Fig. 1:**
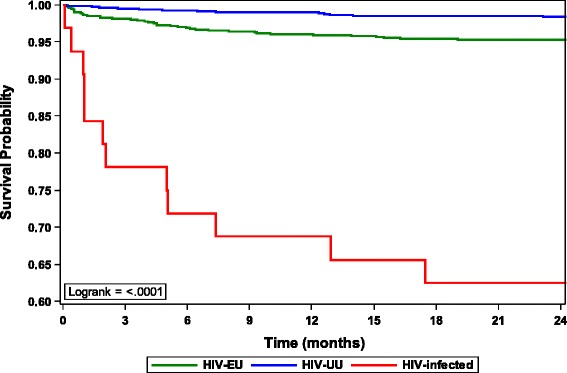
Survival from birth to 24 months among HIV-infected, HIV-exposed uninfected and HIV-unexposed children. Legend: Using the logrank test, there is a significant difference in survival comparing HEU with HUU (*p* < 0.0001) and comparing HIV-infected with HUU (*p* < 0.0001)

Risk factors for child mortality are shown in Table 3. Compared with HU children, the chance of surviving to 24-months was lower among HIV-infected (aHR 21.7, 95%CI 10.3, 46.0) and HEU children (aHR 2.7, 95 % CI 1.6,4.5) (Fig. 1). Sensitivity analyses considering all HIV-exposed children who died with unknown HIV status as infected, or considering them infected if they were high-risk, did not significantly change the effect of HIV-exposure on survival (data not shown). Other factors significantly associated with 24-month mortality among all children in adjusted analyses were maternal death (aHR 8.9, 95%CI 2.1,37.1) and birth during the rainy season (January-March) (aHR 1.8, 95%CI 1.2, 2.6). Although mortality risk among formula fed children was significantly increased when modeled separately from HIV-exposure status (aHR 2.3, 95 % CI 1.5, 3.5), feeding and HIV-exposure status were collinear and could not be modeled together.

**Table 3:** Risk factors for child mortality

	All children (*N* = 3033)		HIV-exposed uninfected children (*N* = 1483)	
Selected risk factors for mortality	Univariate HR (95 % CI)	Adjusted HR (95 % CI)	Univariate HR (95 % CI)	Adjusted HR (95 % CI)
Non-tertiary delivery site	1.1 (0.7,1.5)	–	1.2 (0.7, 1.9)	–
Infant congenital abnormalities	2.5 (0.9,6.8)	1.9 (0.7, 5.4)	4.6 (1.7, 12.5)	5.5 (1.3, 23.8)
Infant birth injuries	1.3 (0.6,2.7)	–	1.8 (0.7, 4.5)	2.7 (0.8, 9.0)
Infant preterm (<37 weeks)	1.4 (0.9,2.3)	1.1 (0.7, 1.8)	1.4 (0.8, 2.4)	1.4 (0.7, 2.9)
Never Breastfed	2.5 (1.7,3.8)	–^a^	1.0 (0.5,1.9)	1.4 (0.5, 3.7)
Introduction of solid food < 6 months	0.7 (0.3,1.4)	–	0.9 (0.3,2.4)	–
Season of birth: Jan–Mar	1.9 (1.3,2.7)	1.8 (1.2, 2.6)	1.6 (1.0,2.5)	1.4 (0.8, 2.8)
Infant HIV-infected^b^	30.0 (15.0,60.0)	21.7 (10.3, 46)	–	–
Infant HIV-exposed^b^	3.5 (2.2,5.5)	2.7 (1.6, 4.5)	–	–
Water not piped directly into home	2.4 (1.2,4.7)	1.8 (0.8, 2.6)	2.1 (0.9. 5.1)	1.6 (0.5, 5.5)
No refrigerator in the home	1.2 (0.8,1.7)	–	1.0 (0.6, 1.6)	–
No/primary maternal education	2.1 (1.1,4.4)	1.5 (0.8, 2.6)	1.8 (1.0, 3.2)	2.7 (1.2, 6.3)
No indoor toilet	1.9 (1.1, 3.2)	1.0 (0.5, 2.0)	1.6 (0.8, 3.3)	0.7 (0.3, 1.9)
Cooking method: Paraffin or woodburning stove	1.4 (1.0,2.1)	1.0 (0.9, 1.6)	1.0 (0.6, 1.6)	–
Maternal Death	9.0 (2.2,36.7)	8.9 (2.1,37.1)	–^c^	–^c^
Maternal Age	1.0 (1.0,1.0)	1.0 (0.9,1..0)	1.0 (1.1)	1.0 (0.9,1.0)
Mother Primiparous	0.6 (0.1,1.0)	0.9 (0.5,1.5)	1.2 (1.7,2.2)	0.8 (0.3,2.1)
Mother Married	0.6 (0.3,1.4)	–	0.5 (0.2,1.6)	0.6 (0.1, 2.5)
Maternal ZDV monotherapy in pregnancy (reference 3-drug ART)	–	–	1.3 (0.8,2.1)	1.1 (0.6, 2.1)
Maternal CD4 < 250 cells/mL	–	–	0.9 (0.4,2.1)	–

^a^Due to collinearity between HIV-exposure status and feeding choice (breast vs. formula feeding), feeding was unable to be added to the multivariate model. When modeled separately, without HIV-exposusre status, the aHR of never breastfeeding was 2.3 (95 % CI 1.5,3.5)

^b^Reference group is HIV-unexposed infants (HU)

^c^Among all HEU infants who died, there were no maternal deaths. Therefore, maternal deaths could not be included as a covariate in the HEU model

In a separate model among only HEU children, independent risk factors for mortality included congenital abnormality (aHR 5.5 95 % CI 1.3, 23.8) and no/primary maternal education (aHR 2.7 95 % CI 1.2, 6.3) (Table 3). The risk of death was non-significantly higher among HEU children who were never breastfed compared with the relatively small number (195) of HEU children who were breastfed for at least a month (aHR 1.4, 95 % CI 0.5, 3.7).

### Population impact of maternal HIV-infection on child mortality

**Fig. 2:**
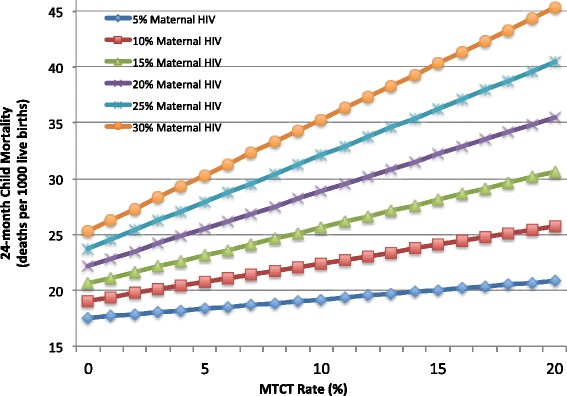
Projected overall childhood mortality in Botswana with varying maternal HIV-infection prevalence and MTCT rate

Applying the cumulative mortality (1.6 % for HU, 4.7 % for HEU and 38 % for HIV-infected) and MTCT rate (2.1 %) to the Botswana population, where 26 % of pregnant women are HIV-infected (Shapiro, RL. Birth Outcomes in Botswana study, unpublished data), over half of under-two mortality is attributable to HIV-exposure (46 % of all deaths expected to be HEUs and 8 % HIV-infected). Figure 2 demonstrates the overall impact of both MTCT rate and maternal HIV prevalence on childhood mortality based on the mortality rates by exposure category determined in this study.

## Discussion

We performed the largest study of 24-month child mortality in Botswana since national scale-up of PMTCT programs. Using cell phone follow up, we were able to achieve a loss-to-follow-up (LTFU) rate of <0.5 % at 24 months. We confirmed previous findings that even if uninfected, HIV-exposed children have decreased survival compared with HIV-unexposed children and found very high mortality among HIV-infected children, with little improvement from the early ART era [5]. These two groups combine to account for more than half of deaths among children under two in Botswana. Mortality among HIV-unexposed children in Botswana was low compared with regional estimates [24], despite breastfeeding durations that fell short of WHO recommendations [25].

Higher mortality among HEU and HIV-infected children may relate to lack of breastfeeding, but our analysis could not untangle the impact of infant feeding method from the impact of HIV-exposure on child survival given extreme collinearity. Although the risk of death for the small number of HIV-exposed breastfed children was lower than those who formula fed, this comparison was underpowered and not statistically significant. We found HIV-infected mothers who breastfed more commonly had markers of lower socioeconomic status compared with HIV-infected mothers who did not breastfeed (data not shown). We would therefore expect HIV-exposed breastfed babies to have higher mortality and so our effect estimate may be biased toward the null. In sum, although we suspect that lack of breastfeeding may have contributed to the higher mortality seen among HEUs, our data were not able to conclusively demonstrate this association.

Mortality among HIV-infected children remains similar to that seen in 2003–2006, near the beginning of the rollout of pediatric ART in Botswana [5]. Our findings were somewhat lower than cumulative 24-month mortality (54 %) reported from a pooled analysis of African countries, most of which had higher overall childhood mortality rates [13]. Previous studies in Botswana have demonstrated very high mortality risk among HIV-infected infants who formula feed from delivery [15], and this was likely a contributor in our cohort as well. Although we did not collect data on ART use among HIV-infected children, we know that children were not initiated prior to diagnosis at their 6-week PCR. Given the emerging data to supporting long-term benefits of early ART initiation [26, 27], our study provides support for HIV testing at birth (in addition to standard six week testing) particularly in areas where formula feeding is common and an HIV diagnosis within days of birth may allow for appropriate counseling to initiate or maintain breastfeeding for these high-risk children.

Infectious diseases were the primary cause of death for all exposure groups, with diarrheal diseases and respiratory diseases most common. Seasonality of birth also appeared to play a role, with children born during the rainy season experiencing higher likelihood of death. The reasons for this association are unknown but could be due to a more contaminated water supply from the rains, food scarcity in the period before the harvest (May/June), or decreased support in the home because family members are often needed for ploughing in this time period. In our study, 88 % of children who died from a cause other than trauma or accident in Botswana had contact with the healthcare system shortly before their death. Therefore, interventions to improve inpatient treatment, recognize at-risk children, and improve diagnostics among those with diarrhea and respiratory symptoms could also improve outcomes.

Our study has several strengths, including a large and geographically diverse sample, 99.5 % follow-up at 24 months and a study design with minimal impact on child outcomes. However, our study likely underestimates early neonatal mortality. All infants enrolled in this study were discharged from the hospital alive and proportionately fewer were very preterm (<37 weeks GA) and very low birthweight (<2500 g) than in the general Botswana population [28, 29]. Although we did not exclude sick infants, mothers of the most critically ill neonates may have been less likely to participate in a study. Extrapolating from other studies of infant outcomes at our study sites [28, 29], we estimate that early in-hospital mortality missed by our study was approximately 1 %; because our sample was otherwise representative, we believe that our mortality estimates may have underestimated overall 24-month mortality in the general population by this amount. Also, while we attempted to control for differences between HIV-infected and HIV-uninfected mothers, unmeasured sociodemographic factors may confound the association between mortality and HIV-exposure status. Our study was also limited in that we were unable to conclusively know the HIV-infection status on most infants who died before their six-week HIV PCR.

## Conclusions

This study highlights the impact of HIV exposure on child mortality in Botswana, even with highly successful and widespread PMTCT and ART programs. Child mortality among HIV-*unexposed* children is low for the region, but achieving this low child mortality for all children in Botswana will require interventions to decrease maternal HIV prevalence and improve outcomes among those who are affected by HIV. Our analysis was unable to determine the direct impact of infant feeding on mortality. However, it seems logical that breastfeeding should be a component of the strategy to improve child outcomes in this setting where most women are on ART and the majority of child deaths are due to diarrhea and pneumonia. Ongoing surveillance to confirm that mortality benefits outweigh the risk from late MTCT among HIV-infected breastfeeding women is warranted.

## Abbreviations

3TC, lamivudine; aHR, adjusted hazard ratio; ART, antiretroviral therapy; BF, breastfeeding; CDC, United States Centers for Disease Control and Prevention; DBS, dried blood spots; ELISA, enzyme linked immunosorbent assay; FF, formula feeding; GA, gestational age; HEU, HIV-exposed uninfected; HIV, human immunodeficiency virus; HU, HIV-unexposed; LTFU, loss to follow up; NVP, nevirapine; PCR, polymerase chain reaction; PMTCT, prevention of mother-to-child transmission of HIV; ZDV, zidovudine

## References

[1] Newell ML, Brahmbhatt H, Ghys PD (2004). Child mortality and HIV infection in Africa: a review. AIDS.

[2] Chopra M, Daviaud E, Pattinson R, Fonn S, Lawn JE (2009). Saving the lives of South Africa's mothers, babies, and children: can the health system deliver?. Lancet.

[3] Mahy, M. Measuring child mortality in AIDS infected countries. Presented at the workshop on HIV/AIDS and Adult mortality in developing countries, Sept 4, 2003. Published online at: http://www.un.org/esa/population/publications/adultmort/UNICEF_Paper15.pdf. Accessed 18 July 2016.

[4] UNICEF/WHO/The World Bank/UN Pop Div. Levels and Trends in Child Mortality. Report 2013. WHO. Botswana neonatal and child health profile. Published online at http://www.who.int/maternal_child_adolescent/epidemiology/profiles/neonatal_child/bwa.pdf. Accessed 18 July 2016.

[5] Thior I, Lockman S, Smeaton LM, Shapiro RL, Wester C, Heymann SJ (2006). Breastfeeding plus infant zidovudine prophylaxis for 6 months vs formula feeding plus infant zidovudine for 1 month to reduce mother-to-child HIV transmission in Botswana: a randomized trial: the Mashi Study. JAMA.

[6] UNAIDS 2013 Progress Report on the Global Plan. Published online at: http://www.unaids.org/sites/default/files/media_asset/20130625_progress_global_plan_en_0.pdf. Accessed 18 July 2016.

[7] Kelly MS, Wirth KE, Steenhoff AP, Cunningham CK, Arscott-Mills T, Boiditswe SC (2015). Treatment failures and excess mortality among HIV-exposed, uninfected children with pneumonia. J Pediatric Infect Dis Soc.

[8] Moraleda C, de Deus N, Serna-Bolea C, Renom M, Quinto L, Menendez C (2014). Impact of HIV exposure on health outcomes in HIV-negative infants born to HIV-positive mothers in Sub-Saharan Africa. J Acquir Immune Defic Syndr.

[9] Landes M, van Lettow M, Chan AK, Mayuni I, Schouten EJ, Bedell RA (2012). Mortality and health outcomes of HIV-exposed and unexposed children in a PMTCT cohort in Malawi. PLoS One.

[10] Koyanagi A, Humphrey JH, Ntozini R, Nathoo K, Moulton LH, Iliff P (2011). Morbidity among human immunodeficiency virus-exposed but uninfected, human immunodeficiency virus-infected, and human immunodeficiency virus-unexposed infants in Zimbabwe before availability of highly active antiretroviral therapy. Pediatr Infect Dis J.

[11] Filteau S (2009). The HIV-exposed, uninfected African child. Trop Med Int Health.

[12] Kuhn L, Kasonde P, Sinkala M, Kanhasa C, Semrau K, Scott N (2005). Does severity of HIV disease in HIV-infected mothers affect mortality and morbidity among their uninfected infants?. Clin Infect Dis.

[13] Newell ML, Coovadia H, Cotrina-Borja M, Rollins N, Gaillard P, Dabis F (2004). Mortality of infected and uninfected infants born to HIV-infected mothers in Africa: a pooled analysis. Lancet.

[14] Taha TE, Hoover DR, Chen S, Kumwenda NI, Mipando L, Nkanaunena K (2011). Effects of cessation of breastfeeding in HIV-1-exposed, uninfected children in Malawi. Clin Infect Dis.

[15] Shapiro RL, Lockman S, Kim S, Smeaton L, Rahkola JT, Thior I (2007). Infant morbidity, mortality, and breast milk immunologic profiles among breast-feeding HIV-infected and HIV-uninfected women in Botswana. J Infect Dis.

[16] Cournil A, De Vincenzi I, Gaillard P, Carnes C, Fao P, Luchters S (2013). Relationship between mortality and feeding modality among children born to HIV-infected mothers in a research setting: the Kesho Bora Study. AIDS.

[17] Fawzy A, Arpadi S, Kankasa C, Sinkala M, Mwiya M, Thea DM (2011). Early weaning increases diarrhea morbidity and mortality among uninfected children born to HIV-infected mothers in Zambia. J Infect Dis.

[18] Rollins NC, Ndirangu J, Bland RM, Coutsoudis A, Coovadia HM, Newell ML (2013). Exclusive breastfeeding, diarrhoeal morbidity and all-cause mortality in infants of HIV-infected and HIV uninfected mothers: an intervention cohort study in KwaZulu Natal, South Africa. PLoS One.

[19] Ásbjörnsdóttir KH, Slyker JA, Maleche-Obimbo E, Wamalwa D, Otieno P, Gichuhi CM (2015). Breastfeeding is associated with decreased risk of hospitalization among HIV-exposed, uninfected Kenyan infants. J Hum Lact.

[20] Walker N, Hill K, Zhao F (2012). Child mortality estimation: methods used to adjust for bias due to AIDS in estimating trends in under-five mortality. PLoS Med.

[21] Statistics Botswana. Health Statistics Report 2009. Available at: http://www.cso.gov.bw/templates/cso/file/File/HealthStatisticsAnnualReport_2009%5B2%5D.pdf. Accessed 18 July 2016.

[22] Ministry of Health, Botswana. 2012 Botswana National HIV and AIDS treatment guidelines. Published online at: http://www.emtct-iatt.org/wp-content/uploads/2013/04/Botswana_National-HIV-AIDS-Guidelines_2012.pdf. Accessed 18 July 2016.

[23] Lawlor DA, Mortensen L, Andersen AM (2011). Mechanisms underlying the associations of maternal age with adverse perinatal outcomes: a sibling study of 264 695 Danish women and their firstborn offspring. Int J Epidemiol.

[24] WHO. Levels and trends in Child mortality 2015. Published online at: http://www.childmortality.org/files_v20/download/IGME%20Report%202015_9_3%20LR%20Web.pdf. Accessed 18 July 2016.

[25] WHO. Guidelines on HIV and infant feeding. 2010. Published online at: http://apps.who.int/iris/bitstream/10665/44345/1/9789241599535_eng.pdf. Accessed 18 July 2016.

[26] Lockman S, Smeaton L, Shapiro R, Thior I, Wester C, Makhema J, et al. Risk factors for and timing of infant mortality among hiv-exposed children in a randomized infant feeding trial in Botswana. Poster presentation, CROI, Boston, Feb 3-8, 2008.

[27] Persaud D, Patel K, Karalius B, Rainwater-Lovett K, Ziemniak C, Ellis A (2014). Influence of age at virologic control on peripheral blood human immunodeficiency virus reservoir size and serostatus in perinatally infected adolescents. JAMA Pediatr.

[28] Zash RM, Souda S, Chen JY, Binda K, Dryden-Peterson S, Lockman S (2015). Reassuring birth outcomes with Tenofovir/Emtricitabine/Efavirenz used for prevention of mother to child transmission of HIV in Botswana. J Acquir Immune Defic Syndr.

[29] Chen JY, Ribaudo HJ, Souda S, Parekh N, Ogwu A, Lockman S (2012). Highly active antiretroviral therapy and adverse birth outcomes among HIV-infected women in Botswana. J Infect Dis.

